# Molecular targets associated with ulcerative colitis and the benefits of atractylenolides-based therapy

**DOI:** 10.3389/fphar.2024.1398294

**Published:** 2024-05-27

**Authors:** Huanzhu Qian, Zhen Ye, Yu Hu, Mingquan Wu, Liulin Chen, Linzhen Li, Zhipeng Hu, Qian Zhao, Chen Zhang, Maoyi Yang, Wen Xudong, Qiaobo Ye, Kaihua Qin

**Affiliations:** ^1^ School of Basic Medical Sciences, Chengdu University of Traditional Chinese Medicine, Chengdu, Sichuan, China; ^2^ Department of Pharmacy, Sichuan Orthopedic Hospital, Chengdu, Sichuan, China; ^3^ State Key Laboratory of Southwestern Chinese Medicine Resources, Chengdu University of Traditional Chinese Medicine, Chengdu, Sichuan, China; ^4^ Department of Gastroenterology, Chengdu Integrated TCM & Western Medicine Hospital, Chengdu, Sichuan, China; ^5^ Health Preservation and Rehabilitation College, Chengdu University of Traditional Chinese Medicine, Chengdu, Sichuan, China

**Keywords:** Atractylodes macrocephala Koidz., atractylenolides, ulcerative colitis, colitis-associated colorectal cancer, natural products

## Abstract

Ulcerative colitis (UC) is a chronic inflammatory disease of the intestines that can significantly impact quality of life and lead to various complications. Currently, 5-aminosalicylic acid derivatives, corticosteroids, immunosuppressants, and biologics are the major treatment strategies for UC, but their limitations have raised concerns. Atractylenolides (ATs), sesquiterpene metabolites found in *Atractylodes macrocephala* Koidz., have shown promising effects in treating UC by exerting immune barrier modulation, alleviating oxidative stress, gut microbiota regulation, improving mitochondrial dysfunction and repairing the intestinal barrier. Furthermore, ATs have been shown to possess remarkable anti-fibrosis, anti-thrombus, anti-angiogenesis and anti-cancer. These findings suggest that ATs hold important potential in treating UC and its complications. Therefore, this review systematically summarizes the efficacy and potential mechanisms of ATs in treating UC and its complications, providing the latest insights for further research and clinical applications.

## Graphical Abstract

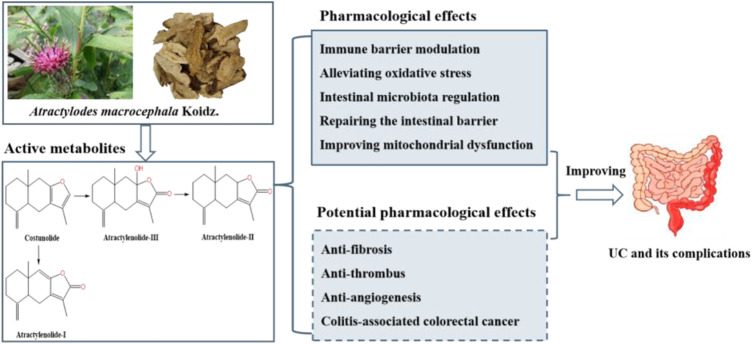

## 1 Introduction

Ulcerative colitis (UC) is a chronic inflammatory bowel disease characterized by continuous and diffuse inflammation in the colonic and rectal mucosa. As its long course, high recurrence, and high risk of malignant transformation, UC has been classified by the WHO as one of the clinically intractable diseases (Li et al., 2022). In Europe and North America, the highest incidence is 24.3/100,000 and 19.2/100,000, respectively. With changing in lifestyle and dietary habits in Asian countries, the incidence has been increasing annually to 7.6/100,000 to 14.3/1000,000, and the morbidity is 2.3/1,000 to 63.6/1,000. The direct and indirect costs associated with UC reach to €1.25 to €2.91 billion annually in Europe and $0.81 to $1.49 billion in the United States. The expensive treatment costs and the gradually increasing incidence have brought about dual challenges of economic burden and public health. Moreover, UC is caused by environmental factors acting on genetically susceptible populations that lead to gut microbiota imbalance or intestinal barrier damage resulting from mucosal immune dysfunction. Symptoms include persistent or recurrent diarrhea, accompanied by abdominal pain urgency, weight loss, mucus bloody stools and other varying degrees of systemic symptoms, lasting more than 4–6 weeks, leading to repeated intermittent acute episodes that may last for years, causing discomfort to patients affected by this disease (Xue et al., 2023). In response to the clinical symptoms and pathological characteristics of UC, clinical treatment is constantly developing and updating, aiming for better treatment of UC and its complications.

Recently, the first-line drugs for the treatment of UC include 5-aminosalicylic acid, corticosteroids, and immunosuppressants (Feuerstein and Cheifetz, 2017). Various formulas and combination therapies of traditional drugs play an important role in treating mild to moderate UC(Le Berre et al., 2023). In recent years, with in-depth research on the pathogenesis of UC, the development of molecularly targeted biologics has become a revolutionary breakthrough in the treatment of UC. The emergence of biologics has greatly alleviated refractory UC that cannot be relieved by traditional drugs and improved the therapeutic effect of UC(Nakase et al., 2022). With the widespread use of these medications, several adverse reactions have gradually been found. Surveys have shown that 30%–55% of patients have no response to molecularly targeted drugs during induction or develop drug resistance in the later stages of treatment. A study has reported that more than 10% of patients have ineffective drug therapy and require surgical treatment (Xu et al., 2023). In addition, due to the recurrent episodes and numerous complications of UC, patients often suffer from great physical, psychological and economic pressure (Burisch et al., 2023). Therefore, the development of effective treatment strategies is essential for the prevention and treatment of UC and its complications.

Natural products are potential therapeutic strategies for UC in clinical practice (Lu et al., 2023). *Atractylodes macrocephala* Koidz., one of the commonly used traditional Chinese medicines in clinic (Yang et al., 2021), is widely applied to treat digestive system diseases due to its effects of invigorating the spleen and replenishing qi, drying dampness and promoting diuresis, stopping sweating and calming the fetus (Zhou et al., 2021). Studies have shown that *A. macrocephala* Koidz. plays an important role in the treatment of UC(Feng et al., 2018; Deng et al., 2019a; Deng et al., 2019b; Chen W. et al., 2021; Yu et al., 2022; Zou and Zhu, 2022). Atractylenolides (ATs) are sesquiterpene compounds extracted from the *Atractylodes* genus of the Asteraceae family, which is one of the main active ingredients in *A. macrocephala* Koidz. (Wang Y. M. et al., 2021).

ATs have a variety of pharmacological effects and significant therapeutic effects on inflammatory diseases. In addition, ATs can be rapidly absorbed and metabolized slowly, making them valuable for drug development (Deng et al., 2021). ATs have various configurations, among which AT-I, AT-II and AT-III are extensively studied configurations with strong pharmacological effects. In recent years, many scholars have paid high attention to the pharmacological activities of these three types of ATs (Yang et al., 2021).

Studies have shown that ATs can effectively treat UC through their immune barrier modulation and alleviating oxidative stress, as well as intestinal barrier repair, improvement of mitochondrial dysfunction, and regulation of the intestinal microbiota (Han et al., 2022; Qu et al., 2022). Additionally, it also has the effects of anti-thrombus, reducing multiorgan fibrosis, and decreasing pathological angiogenesis (Yang et al., 2021). These reports suggest that ATs have great advantages in the treatment of UC and its complications. However, the targets of ATs for the treatment of UC and their underlying biological processes remain to be comprehensively reviewed. The aim of this paper is to comprehensively summarize the efficacy and potential mechanisms of ATs for the treatment of UC and its complications and to provide a scientific basis for the in-depth study and clinical application of ATs. Tables 1, 2 summarize the applications of ATs in cellular and animal experiments, respectively.

**TABLE 1:** Use of ATs in the prevention and treatment of UC at the cellular level.

ATs	Cells	Modeling methods	Dose and duration	Effects	Mechanisms	Ref.
AT-Ⅰ	RAW264.7 macrophages	LPS	25, 50, 100 μM	MD-2, CD14, SR-A, MyD88, TNF-α, IL-6, ERK1/2, p38↓	TLR4, NF-kB↓	Ji et al. (2014)
	peritoneal macrophages	LPS	1–100 µM for 24h	TNF-α, NO, iNOS↓	TLR4↓	Li et al. (2007b)
	IEC-6(CRL 1592)	wounding	5 μM, 10 μM for 8 h	Cell migration and proliferation↑; polyamines content↑; TRPC1, PLC-γ1↑	Ca^2+^↑	Song et al. (2017)
AT-Ⅱ	IEC-6	wounding	0–160 μM for 24h and 48h	Cell proliferation and migration↑; IL-2, IL-10, ODC↑; STIM1, STIM2, TRPC1, RhoA↑	Ca^2+^↑	Ren et al. (2021b)
AT-Ⅲ	RAW264.7 macrophages	LPS	1–100 µM	NO, PGE2, TNF-α, IL-6↓	TLR4/NF-kB/MAPK↓	Wang et al. (2019a)
	peritoneal macrophages	LPS	1–100 µM for 24h	TNF-α, NO, iNOS↓	TLR4↓	Li et al. (2007b)
	Human mast cells	PMACI	1 uM; 10 uM; 100 uM	IL-6, IL-1β, p38, JNK↓	caspase-1/RIP-2/NF-κB↓	Kang et al. (2011)
	Human mast cells	TSLP	1 uM; 10 uM; 100 uM	IL-6, IL-1β, TNF-α, IL-13, IL-8↓; Bcl2, procaspase-3↓; caspase-3, cleaved PARP↑; mast cell proliferation↓	pSTAT6, pSTAT5, pSTAT3↓	Yoou et al. (2017)
	IEC-6	LPS	80 *μ*M for 24 h; 40 or 80 *μ*M for 12 h	occludin, ZO-1↑; mtDNA, MMP, complex I, complex IV↑	pAMPK/SIRT1/PGC-1α↑	Han et al. (2022)
	IEC-6	wounding	0–160 μM for 24h and 48h	Cell proliferation and migration↑; IL-2, IL-10, ODC↑; STIM1, STIM2, TRPC1, PLC-γ1, RhoA↑	Ca^2+^↑	Ren et al. (2021b)
	IEC-6	TGF-β1	1, 10, 20 μmol/L for 24h	EMT, vimentin, N-cadherin↓; E-cadherin, ZO-1↑	AMPK↑	Huang et al. (2022)

**TABLE 2:** Animal experiments on use of ATs to treat UC.

ATs	Animals	Modeling methods	Dose and duration	Effects	Mechanisms	Admini-stration	Positive control	Ref.
AT-Ⅰ	Male BALB/c mice	DSS	25 mg/kg; 50 mg/kg; for 7 days	TNF-α, IL-6, IL-1β↓; MUC2, zo-1, occludin↑; diversity and abundance of intestinal flora↑	*SPHK1*/PI3K/AKT↓; *SPHK1, B4GALT2*↓	Oral gavage	SASP 250 mg/kg/day	Qu et al. (2022)
	Mice (intestinal dysbiosis)	ampicillin, vancomycin, neomycin, and metronidazole	NA	abundance of *Lactobacillus* and *Bacteroides*↑; abundance of *Escherichia* and *Candidatus*↓; Bcl-2 and Bcl-xL↓	TLR4/MyD88/NF-kB↓	Oral gavage	NA	Ren et al. (2021b)
	Shen Zhu capsule	System pharmacology	NA	IL-6, TNF-α, INF-γ, IL-1β, COX-2↓	TLR4/MyD88/NF-kB↓	NA	NA	Feng et al. (2018)
AT-Ⅲ	Male C57BL/6J mice	DSS	5 mg/kg; 10 mg/kg; for 7days	TNF-α, IL-6, COX-2, iNOS, MPO, MDA↓; GSH, SOD, occludin, ZO-1↑	pAMPK/SIRT1/PGC-1α↑	inject through tail vein	SASP 200 mg/kg/day	Han et al. (2022)
	mice	TNBS	5 mg/kg; 10 mg/kg; 20 mg/kg; for 7days	IL-1β, TNF-α↓; myeloperoxidase activity↓; ROS, MDA↓; SOD, CAT, GPx, GR↑; Regulating intestinal microbiota	FPR1/Nrf2↓	Oral gavage	NA	Ren et al. (2021a)
	mice	DSS	5 mg/kg; 10 mg/kg	mitochondrial dysfunction↓	pAMPK/SIRT1/PGC-1α↑	inject through the tail vein	SASP 200 mg/kg/day	Han et al. (2022)

Note: “NA” represents Not Available.

## 2 Biological characteristics of ATs

### 2.1 Sources of ATs

ATs have a wide range of sources. The dried rhizome of *A. macrocephala* Koidz., a plant belonging to the Asteraceae family (Wang Y. M. et al., 2021), is the major source of ATs. Plants of the *Atractylodes* genus are mainly distributed in eastern Asia with seven subspecies, namely, *A. macrocephala* Koidz., *Atractylodes japonica* Koidz. ex Kitam., *Atractylodes lancea* (Thunb.) DC., *Atractylodes chinensis* (DC.) Koidz., *Atractylodes carlinoides* (Hand. Mazz.) Kitam., *Atractylodes coreana* (Nakai) Kitam. and *A. lancea* (Thunb.) DC. Subsp. Luotianensis (Liu et al., 2016).

The commonly used clinical Atractylodes are the rhizomes of the *A. lancea* (Thunb.) DC. and *A. chinensis* (DC.) Koidz. (Wang Y. M. et al., 2021), which are also important sources of ATs. Furthermore, *Codonopsis pilosula* from the Campanulaceae family is also an important source of ATs. The existence of ATs can be used as an important identification and quality evaluation index of *C. pilosula* (Wang et al., 2023). Various plants in the Orchidaceae family, such as *Cremastra appendiculata* also contains ATs (Deng et al., 2021) (Table 3).

**TABLE 3:** Sources of ATs in the plants of *Atractylodes* genus.

Sources	Metabolites
*A. macrocephala* Koidz	AT-Ⅰ, AT-Ⅱ, AT-Ⅲ, AT-Ⅳ, AT-Ⅴ, AT-Ⅵ, AT-Ⅶ
*A. lancea* (Thunb.) DC.	AT-Ⅰ, AT-Ⅱ, AT-Ⅲ
*A. chinensis* (DC.) Koidz	AT-Ⅰ, AT-Ⅱ, AT-Ⅲ
*A. japonica* Koidz. ex Kitam	AT-Ⅰ, AT-Ⅱ, AT-Ⅲ

### 2.2 Classification of ATs

ATs belong to the sesquiterpene class of metabolites, which are considered one of the major active metabolites in *Atractylodes* genus plants. The common sesquiterpenes found in *A. macrocephala* Koidz. include atractylenolide, atractylon, AT-I, AT-II, AT-III, AT-IV, diacetyl-atractylodinolide, AT-V, AT-VI, AT-VII, 3β-acetoxyatractylenolide, dehydroatractylonolide, isoatractyloside A, atractylodinamide and 8β-methoxy-atractylenolide II (Yao et al., 2019). Furthermore, in recent years, a number of new sesquiterpenoids have been isolated from the rhizomes of *A. macrocephala* Koidz., such as atractylenolactam A, atractylenolactam B, 8-methoxy-atractylenolide V and 15-acetoxyl atractylenolide III (Hoang et al., 2016; Wang et al., 2022; Hai et al., 2023). Common sesquiterpenoids in *A. lancea* (Thunb.) DC. include β-eudesmol, acoriol, atractylon, AT-I, AT-II and AT-III (Cho et al., 2016; Zhao et al., 2022). AT-I, AT-II and AT-III are important active metabolites shared by *A. macrocephala* Koidz., *A. lancea* (Thunb.) DC., *A. chinensis* (DC.) Koidz. and *A. japonica* Koidz. ex Kitam. ATs are being extensively studied due to their wide range of pharmacological activities. Therefore, this paper primarily summarizes the therapeutic effects and potential mechanisms of AT-I, AT-II and AT-III in UC.

### 2.3 Structures of ATs

**FIGURE 1:**
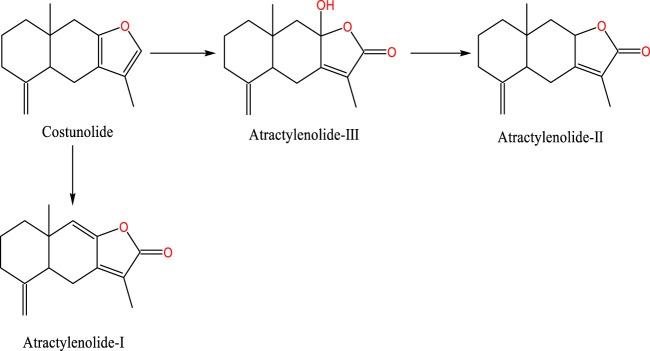
Chemical structural formulae of ATs.

As a shared precursor of sesquiterpenoid metabolites, cis-farnesol undergoes an acetyl-CoA reaction to yield farnesyl pyrophosphate, which is further converted into farnesyl caryophyllene. Under the catalytic influence of sesquiterpene cyclase, farnesyl caryophyllene is transformed into sesquiterpenes. These sesquiterpenes experience double bond cleavage and condensation to form intermediates, which subsequently undergo oxidation and dehydration to generate costunolide. Costunolide, being inherently unstable, self-oxidizes to form a diene diol structure. This structure, following varying degrees of oxidation and double bond isomerization, cyclizes to form AT-I, AT-III or couples to create bisabolene lactone. Furthermore, AT-III can be dehydrated to produce AT-II (Yao et al., 2019). The study on cytochrome P450 (CYP450) simulated the oxidation model of mutual transformation for three types of ATs. AT-II can be oxidized to generate AT-III, and it can also be converted to AT-I by dehydration (Kim et al., 2018) (Figure 1).

## 3 Pharmacokinetics

### 3.1 AT-Ⅰ

Studies on the absorption of AT-I in the intestines of rats revealed that it is a highly permeable metabolites that can be rapidly absorbed throughout the intestine. The absorption characteristics of AT-I in different intestinal segments of rats were analyzed by an *in vivo* intestinal circulation model and high-performance liquid chromatography. There was no significant difference in the absorption rate constant of AT-I in duodenum, jejunum, ileum and colon. The passive diffusion mediated by the P-glycoprotein (Pgp) efflux transport system facilitated its absorption and distribution (Wang et al., 2009a). After oral administration of AT-I in rats at a dose of 50 mg/kg, the peak time (T_max_), maximum concentration (C_max_), area under the curve (AUC), and absorption rate constant (Ka) were 0.37 ± 0.19 h, 0.26 ± 0.05 μg/mL, 1.95 ± 0.30 μg h/mL and 10.08 ± 5.60 h-1, respectively (Wang et al., 2009a; Li et al., 2012).

In addition, rats liver mitochondria were co-cultured with AT-I, and the bio-transformation products were characterized after pretreatment with phenobarbital sodium. The results show that the main metabolites of AT-I were hydroxylated and methylated products, and the C-1, C-2, and C-3 positions of ring A could be specifically oxidized. Therefore, the reaction between AT-I and reactive oxygen species (ROS) may be one of the main mechanisms by which AT-I exerts its anti-inflammatory and anti-cancer effects to prevent or alleviate oxidative damage caused by ROS to bio-molecules (Li and Yang, 2013).

### 3.2 AT-Ⅱ

The pharmacokinetic study conducted on rats after intragastric administration of AT-II showed that the elimination half-life (T1/2) and Tmax of the drug were 0.14 h and 0.41 h, respectively, indicating that AT-II was rapidly absorbed in rats. The study also showed that after oral administration of *A. macrocephala* Koidz. extract equivalent to a dose of 82.82 μg/kg of AT-II in rats, the C_max_, AUC0→12, clearance rate (CLz/F) and T1/2 values of AT-II were 7.99 ± 0.90 ng/mL, 28.46 ± 7.71 ng h/mL, 0.043 ± 0.015 L/(kg•h) and 2.63 ± 1.08 h, respectively (Shi et al., 2012). When co-incubated with rat liver microsomes, AT-II mainly underwent hydroxylation and epoxidation reactions at the double bonds between C-4 and C-15, as well as at positions C-1, C-3, C-8, and C-13, which are easily hydroxylated (Li et al., 2013).

### 3.3 AT-Ⅲ

AT-Ⅲ has been reported as a compound with rapid absorption characteristics. After oral administration of AT-III to SD rats at a dose of 100 mg/kg, the results showed that AT-III was rapidly absorbed and distributed evenly in the body (Li et al., 2006). After administering an extract of *A. macrocephala* Koidz. equivalent to a dose of 185.16 μg/kg of AT-III by gavage in rats, the C_max_, AUC 0→12, CLz/F, and T1/2 values of AT-III were 9.79 ± 1.79 ng/mL, 37.43 ± 7.86 ng h/mL, 0.029 ± 0.0068 L/(kg•h) and 4 ± 1.94 h, respectively (Shi et al., 2012). High-performance liquid chromatography showed that it was mainly excreted through the spleen, followed by liver and kidney. The metabolism pathways of AT-III encompass various processes such as methylation, oxidation, hydroxylation, dihydroxylation, reduction, glycosylation, sulfation, glucuronic acid conjugation, cysteine and N-acetylcysteine binding, among others. After oral administration of AT-III, more than 50 metabolites were identified in rat feces urine and plasma (Jiang et al., 2019).

Current studies have shown that AT-I, AT-II, and AT-III can be quickly absorbed into the blood and detected within 0.0833 h, and the retention time in the plasma is about 12 h. The inverted intestinal sac model indicated that ATs had similar pharmacokinetic characteristics and could be passively transported by all intestinal segments. However, AT-III had the highest absorption rate in all intestinal segments, and the duodenum was the main site for the absorption of AT-II, while AT-I was absorbed rapidly within entire enteric. ATs are considered to be rapidly absorbed without obvious interaction with each other, suggesting good bioavailability based on their pharmacokinetic data (Gao et al., 2018).

Although changes in experimental factors can alter the pharmacokinetic parameters of ATs, they are rapidly absorbed into the blood and slowly eliminated in rats. Furthermore, there have been no reports of toxic side effects from ATs in the clinic. Therefore, ATs show potential for further drug development. Meanwhile, as key metabolites of traditional Chinese medicines *A. macrocephala* Koidz. and *A. lancea* (Thunb.) DC., the new drug development of ATs holds significant social value in promoting the utilization and development of these botanical drugs.

## 4 The pharmacological activities of ATs

### 4.1 Immune barrier modulation

Although the etiology of UC remains incompletely elucidated, recent advancements underscore the pivotal role of cytokines and immune cells in the pathogenesis of UC (Neurath, 2014). Within adaptive immunity, the dysregulation of Th1/Th2 and Th17/Treg cells is considered an important reason for the immunological imbalance observed in UC (Geremia et al., 2014; Iboshi et al., 2017). Cohort studies have also highlighted substantial dysregulation in B cell responses in UC, underscoring the potential involvement of humoral immunity in the pathogenesis of this condition (Uzzan et al., 2022). Furthermore, the innate immune system of the mucosa has been extensively investigated. Neutrophils, monocytes, and macrophages collectively form the innate barrier of phagocytic cells, playing a critical role in the pathogenesis of UC (Zhou and Liu, 2017; Uhlig and Powrie, 2018). Consequently, the modulation of the balance of immune cells and cytokines to restore intestinal mucosal homeostasis represents a primary therapeutic target in the clinical management of UC (Tong et al., 2021).

AT-I and AT-III exhibited pronounced immune barrier modulation effects *in vivo* and vitro. In a murine model of DSS (Dextran Sulfate Sodium Salt)-induced UC, AT-I and AT-III markedly suppressed the production of pro-inflammatory cytokines such as TNF-α, IL-6, cyclooxygenase-2 (COX-2) and iNOS, ameliorating colonic inflammation response (Han et al., 2022; Qu et al., 2022). In a study involving LPS (Lipopolysaccharide)-induced murine macrophages RAW264.7, AT-I, and AT-III shown no inhibitory effect on cell proliferation within the concentration range of 1 μM–100 μM. They effectively inhibited the production of NO, PGE2, TNF-α and IL-6. Their immune barrier modulation effects may be associated with the suppression of the expression of NF-kB, ERK1/2, and p38 (Ji et al., 2014; Ji et al., 2016). Furthermore, research indicates that AT-I exhibits a more pronounced inhibitory effect on the activation of macrophages, leading to the production of TNF-α and NO in response to LPS, compared to AT-III (Li et al., 2007b). Toll-like receptor 4 (TLR4) binding to its ligand, LPS, triggers MyD88 protein adapter-mediated inflammatory responses, playing a pivotal role in the development of UC. Activation of signaling molecules downstream of TLR4, including NF-kB and those mediated by MAPK kinases such as JNK, ERK and p38, results in the transcription of numerous pro-inflammatory genes, including TNF-α, IL-6, IL-1β, and COX-2, subsequently eliciting a UC inflammatory response (Wang G. et al., 2019). Therefore, AT-I and AT-III act as antagonists of the TLR4 receptor and effectively suppress the release of pro-inflammatory cytokines by inhibiting the TLR4/NF-kB/MAPK signaling pathway, leading to a significant improvement in UC.

The systematic pharmacological investigation of the ginsenoside capsule also yielded consistent results. One of the ginsenoside capsule’s bioactive ingredients, AT-I, has been shown to dramatically decrease tissue levels of pro-inflammatory cytokines such IL-6, TNF-α, and INF-γ. Its mechanism is associated with the inhibition of NF-kB activation mediated by the MyD88 protein adapter triggered by the TLR4 receptor, resulting in the transcriptional downregulation of numerous pro-inflammatory genes, including TNF-α, IL-6, IL-1β, and COX-2, thereby mitigating the inflammatory response (Feng et al., 2018). Additionally, a study revealed that AT-I effectively inhibits increased vascular permeability in acetic acid-induced mice and opposes granulation tissue proliferation, indicating therapeutic effects of AT-I on both acute and chronic inflammation (Li et al., 2007a).

Mast cells originate from precursor cells in the bone marrow, serving as the first line of defense in the immune system against various challenges. In a murine model of UC, histamine derived from mast cells mediates neutrophil infiltration into the colonic mucosa through H4R, participating in the inflammatory response. This suggests that mast cells serve as alternative therapeutic targets beyond adaptive immunity (Wechsler et al., 2018). Additionally, compared to non-inflammatory UC regions, research on human colonic tissue show a considerable upregulation of certain mast cell mediators in inflammatory UC regions. Variants that reduce mast cell activity effectively prevent the development of UC (Chen E. et al., 2021). AT-III possesses the capability to reduce mast cell proliferation induced by thymic stromal lymphopoietin (TSLP) and the production of pro-inflammatory cytokines, including IL-6, IL-1β, TNF-α and IL-8 (Yoou et al., 2017). Additionally, AT-III may suppress immune responses by inhibiting the secretion of IL-6 within mast cells (Kang et al., 2011). Therefore, serving as a multifunctional immunoregulator, AT-III plays a crucial role in regulating both innate and adaptive immunity.

### 4.2 Alleviating oxidative stress

In the pathological processes of UC, inflammation and oxidative stress are believed to play pivotal roles, exacerbating the immune response and intestinal damage of UC (Maloy and Powrie, 2011; Zhu and Li, 2012). Chronic inflammation serves as a significant stimulant for the overproduction of ROS (Santhanam et al., 2012), and high concentrations of ROS can damage cellular structures, leading to secondary mucosal injury (Ho et al., 2018). This may contribute to the perpetuation and consolidation of intestinal inflammation in UC. Furthermore, such damage increases the risk of pathogen invasion, which may trigger new immune responses, thereby exacerbating the progression and chronic damage associated with UC. Therefore, anti-inflammatory and anti-oxidant approaches are vital in the clinical treatment of UC.

Treatment with AT-III for UC mice by TNBS-induced for 14 days yielded promising results. AT-III administered at high and medium doses resulted in a considerable improvement in both histopathological damage and symptoms. Also, pro-inflammatory cytokines like TNF-α and IL-1β were downregulated. Additionally, the activity levels of myeloperoxidase were attenuated. AT-III also reduced the expression of pro-oxidative markers, ROS, and malondialdehyde (MDA) in UC mice while enhancing the expression levels of endogenous anti-oxidants, including catalase (CAT), superoxide dismutase (SOD), and glutathione peroxidase (GPx), among others. Further investigations suggested that AT-III may exert its anti-oxidative effects through the modulation of formy1 peptide receptor 1 (FPR1) and nuclear factor erythroid 2-related factor 2 (Nrf2) pathways (Ren et al., 2021a).

Nrf2 is a pivotal transcription factor associated with cellular anti-oxidant responses and serves as the central regulator for maintaining cellular redox homeostasis (Wang R. et al., 2019). Under physiological conditions, Nrf2 resides in the cytoplasm and forms a complex with Kelch-like ECH-associated protein 1 (Keap1), subject to proteasomal degradation, maintaining an inhibitory state. Upon exposure to oxidative stress, Nrf2 dissociates from the Keap1/Nrf2 complex, translocates into the nucleus, forms a heterodimer with small Maf proteins, and binds to anti-oxidant response elements (ARE), thereby activating downstream anti-oxidant proteins such as heme oxygenase-1 (HO-1) to exert anti-oxidative and anti-inflammatory effects (Lin et al., 2020). The extract of *A. macrocephala* Koidz. was regarded as ideal HO-1 expression promoter, which has better efficacy than sulfasalazine drugs for preventing UC recurrence (Han et al., 2017). There are reports that both AT-II and AT-III have similar anti-oxidant effects that activate the Nrf2 pathway as anti-oxidants (Yang et al., 2017).

### 4.3 Intestinal microbiota regulation

While the pathogenesis of UC is still under investigation, the prevailing consensus among researchers suggests that environmental factors, in conjunction with the intestinal microbiota, act on genetically susceptible individuals, triggering immune-inflammatory responses that disrupt the intestinal barrier and lead to the development of UC (Mirkov et al., 2017). The human intestinal tract harbors a diverse microbiome comprising various bacteria, viruses, and phages. Dysbiosis in the intestinal microbiota can promote the development of UC by increasing the pathogenic bacterial load, reducing the levels of beneficial bacteria, and disrupting normal immune tolerance. It is currently established that UC patients exhibit abnormalities in the composition of their intestinal microbiota, potentially involving the reduction in overall diversity and the expansion of pathogenic strains (Ni et al., 2017). Therefore, the management of intestinal dysbiosis is of paramount importance in the comprehensive treatment of UC.

Results from 16S sequencing of fecal samples from UC experimental mice indicate that AT-Ⅰ increases the diversity and abundance of the intestinal microbiota in UC mice. Further investigations reveal that AT-Ⅰ suppresses inflammation through the SPHK1/PI3K/AKT axis and modulates fructose and lactose-related metabolism by targeting two genes (*SPHK1* and *B4GALT2*). This regulation influences the composition of the intestinal microbiota and ameliorates colonic inflammation (Qu et al., 2022). Moreover, a study comparing changes in the gut microbiota of mice following AT-I treatment, utilizing a mouse model of intestinal dysbiosis, demonstrates that AT-I adjusts the gut microbiota by increasing the abundance of beneficial bacteria such as lactobacilli and bifidobacteria, while dose-dependently reducing the abundance of harmful bacteria like *Escherichia coli* and *Candida* (Liu et al., 2021). This work provides more evidence that the gut microbiota mediates the inhibitory effect of AT-Ⅰ on tumor growth, with possible mechanisms involving the downregulation of the TLR4/MyD88/NF-kB signaling pathway.

Researchers have also conducted fecal 16S DNA analysis in TNBS-induced mice following AT-III treatment. The data suggests that AT-III effectively ameliorates the reduction of beneficial microbial communities at the genus level in UC mice induced by TNBS, which alters the structure and composition of the gut microbiota. Among these changes, there is a significant increase in lactobacilli, which can modulate the expression level of FPR1 and subsequently regulate the oxidative stress levels in the colorectal tissue (Ren et al., 2021a). Thus, AT-III regulates oxidative stress via the FPR1 and Nrf2 pathways, impacting the gut microbiota’s growth and reducing the TNBS-induced colonic inflammatory response. (Table 4).

**TABLE 4:** Changes in intestinal microbiota after treatment of ATs.

ATs	Model	Increased intestinal microbiota	Decreased intestinal microbiota
AT-I Treatment	Intestinal dysbiosis mouse model	*Lactobacillus*; *Bacteroides*	*Escherichia*; Candidatus
AT-I Treatment	DSS	Firmicutes phylum; *Lactobacillus* genus.; Erysipelatoclostridium genus; Lachnospiraceae genus	Proteobacteria phylum; *Helicobacter* genus; *Shigella* genus; Rodentibacter genus; *Enterobacter* genus
AT-III Treatment	TNBS	Bacteroidetes; *Lactobacillus*; *Staphylococcus*	Actinobacteria; Oscillospira

### 4.4 Repairing the intestinal barrier

The histopathological features of UC encompass epithelial ulceration, infiltration of lamina propria immune cells, crypt abscesses, splenomegaly and hepatomegaly, and compromised intestinal barrier function (Xu et al., 2023). The integrity of the intestinal epithelium is a critical determinant affecting the function of the gut barrier. The migration and proliferation of intestinal epithelial cells (IEC) represent fundamental mechanisms for mucosal ulcer healing and wound repair. AT-I promotes the migration and proliferation of IEC-6 cells, which is possibly mediated through the augmentation of cytoplasmic calcium levels, driven by polyamines (Song et al., 2017). Previous research has also shown that both AT-I and AT-III can reverse the reduced expression levels of mucin MUC2 and tight junction proteins (zo-1, occludin) in the colonic tissues of UC mice (Han et al., 2022; Qu et al., 2022). Therefore, AT-I and AT-III can enhance the expression of tight junction proteins, effectively promoting the repair of intestinal epithelium and ameliorating the mucosal barrier function in UC.

The research on the role of *A. macrocephala* Koidz. extract in intestinal epithelial repair indicates that AT-II and AT-III exert varying degrees of promotion on IEC-6 cell proliferation and migration. AT-III stands out as the primary active ingredient in *A. macrocephala* Koidz. Extract, showing its ability to stimulate cell proliferation. The combination of ATs exhibits superior effectiveness in promoting intestinal epithelial repair (Ren et al., 2021b). *In vivo* studies showed that giving Wistar rats 10 mg/kg of AT-III dramatically reduced the amount of ethanol-induced stomach ulcers. AT-III achieves this by upregulating tissue metalloproteinase inhibitors, consequently inhibiting the expression of matrix metalloproteinases, specifically MMP-2 and MMP-9, within the ulcerated gastric tissue (Wang et al., 2010). Hence, a combined utilization of components such as AT-I, AT-II, and AT-III can be considered when promoting intestinal epithelial repair.

### 4.5 Improving mitochondrial dysfunction

Research indicates that patients with UC may possess an inherent susceptibility to mitochondrial dysfunction, which can be masked by the intestinal environment (Ho and Theiss, 2022). Mitochondrial dysfunction leads to the production of high levels of superoxide and hydrogen peroxide within cells. These substances can combine with iron to generate free radicals, causing oxidative damage to macromolecules within the mitochondria, with mitochondrial DNA being particularly vulnerable (Shimada et al., 2012). Mitochondrial damage results in energy deficits that increase the susceptibility to cell death while simultaneously exacerbating the inflammatory response within the UC.

**FIGURE 2:**
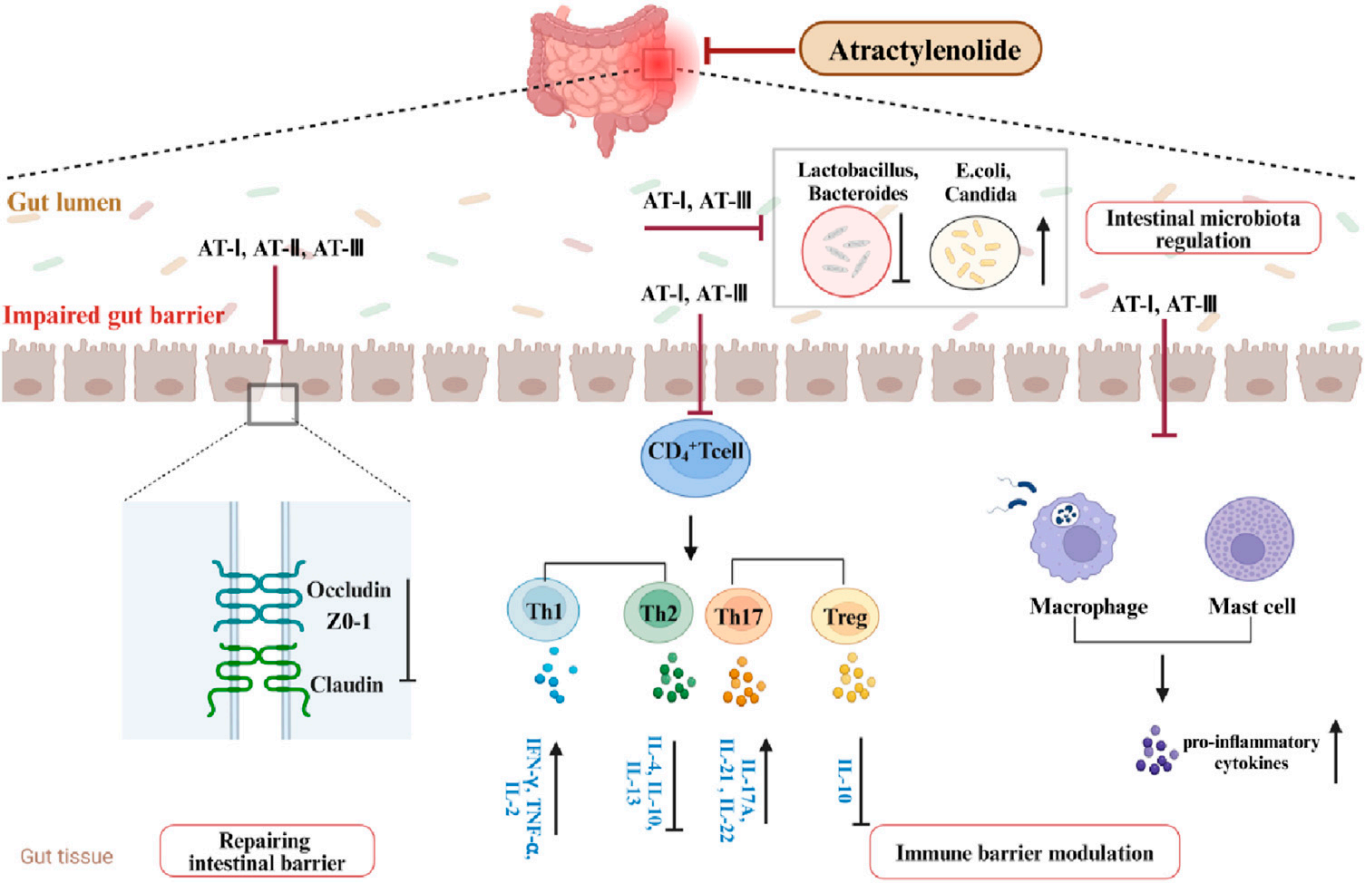
Pharmacologic effects of ATs in ameliorating UC.

**FIGURE 3:**
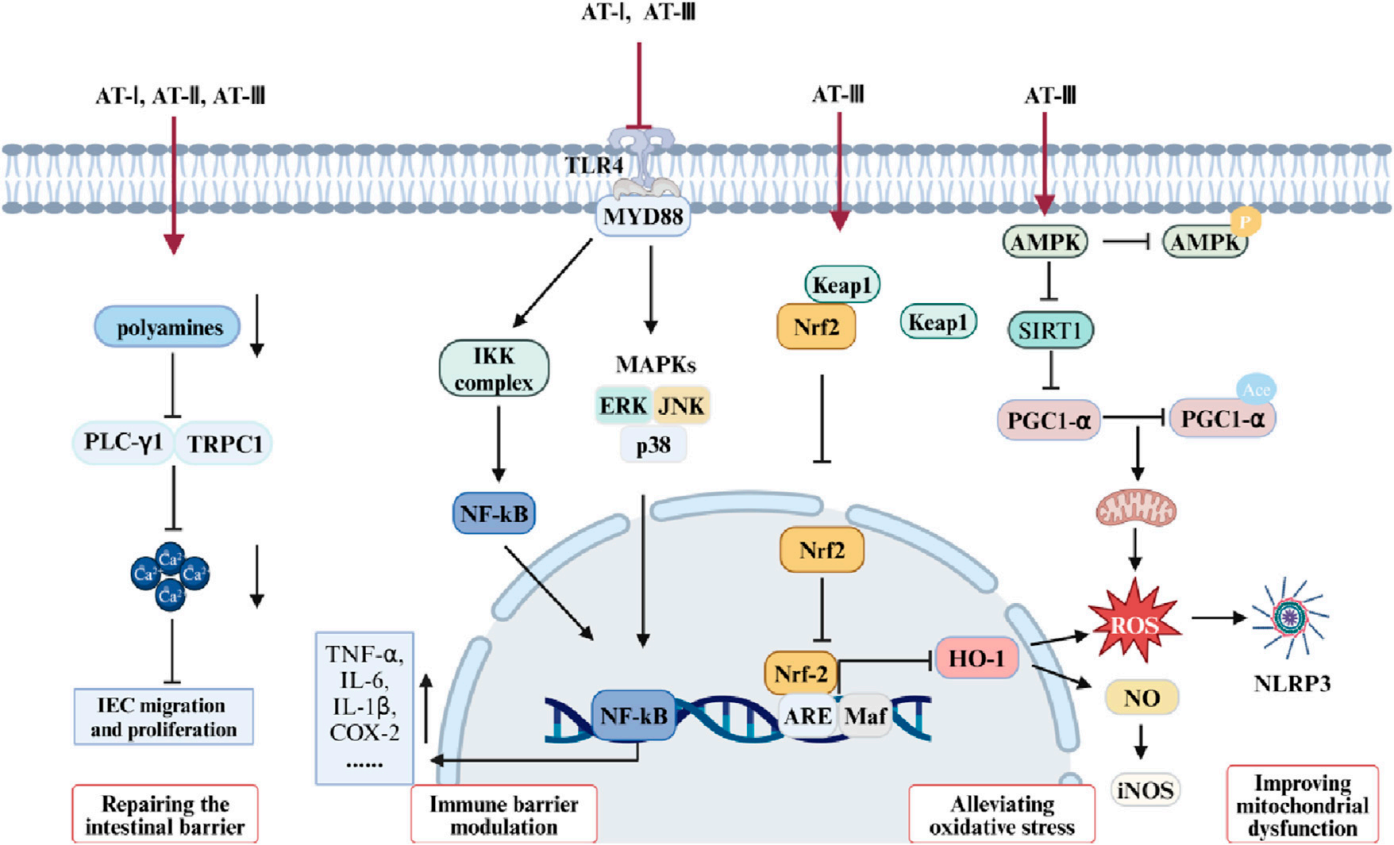
Mechanisms of ATs in the treatment of UC.

Receptor-γ coactivator 1-α (PGC-1α) is prominently expressed in the uppermost intestinal epithelial cells, furthest from the crypt base. It drives mitochondrial biogenesis and the metabolic shift towards mitochondrial respiration (D'Errico et al., 2011). Research suggests that reduced PGC-1α in intestinal epithelium is associated with mitochondrial dysfunction, epithelial barrier damage, and inflammatory responses in UC. Studies have indicated that enhanced PGC-1α deacetylation can repair damaged mitochondria and preserve intestinal barrier function (Cunningham et al., 2016). AT-III was reported to alleviate mitochondrial dysfunction in DSS-induced colitis mice, consistent with results from LPS-treated IEC-6 cells. Further mechanistic studies reveal that AT-III activates AMP-activated protein kinase (AMPK) and sirtuin 1 (SIRT1), subsequently increasing PGC-1α expression and promoting its deacetylation (Han et al., 2022). Therefore, AMPK/SIRT1/PGC-1α may represent a potential pathway through which AT-III ameliorates mitochondrial dysfunction in the UC intestinal epithelium. Studies suggest that mitochondria play a crucial role in various cellular functions, rapidly responding to extracellular stimuli and cellular demands while dynamically communicating with other organelles (Ni et al., 2015). Maintaining normal mitochondrial function may improve inflammatory responses, oxidative stress, and intestinal epithelial barrier damage in UC (Yeganeh et al., 2018; Wang S. Q. et al., 2019) (Figures 2, 3).

### 4.6 Anti-fibrosis

Intestinal fibrosis is considered a common pathological feature of UC and represents a long-term complication characterized by excessive proliferation of myofibroblasts and collagen deposition (Rieder et al., 2017; Laudadio et al., 2022). Under normal circumstances, damaged colonic tissue undergoes a healing process through the intestine. However, if the intestine continues to sustain damage, it can result in chronic intestinal inflammation, marked by persistent injury and repair, ultimately leading to intestinal fibrosis (Wang R. et al., 2021). Intestinal fibrosis can result in frequent luminal narrowing, severely compromising the physiological function of the intestine and significantly impacting the quality of life (Schwab et al., 2019). Current evidence suggests that persistent inflammation is an important factor leading to fibrosis, but anti-inflammatory treatment has not fully reduced the overall incidence of intestinal fibrosis (Abraham et al., 2017; Henderson et al., 2020). Therefore, anti-fibrosis drugs are necessary to be developed.

Research suggests that up-regulating the expression of Nrf2 to inhibit the TGF-β1/Smad pathway can ameliorate inflammation-related intestinal fibrosis, underscoring the potential therapeutic targeting of Nrf2 in alleviating intestinal fibrosis (Wang R. et al., 2021). Previous studies have indicated that AT-III serves as an effective activator of the Nrf2 transcription factor (Zhu and Li, 2012). Therefore, AT-III is anticipated to play a role in anti-intestinal fibrosis through the Nrf2/TGF-β1/Smad pathway. Another study also suggests that AT-III can inhibit epithelial mesenchymal transition (EMT) in IEC-6 cells by activating the AMPK signaling pathway, thereby suppressing cell migration induced by TGF-β. Furthermore, AT-III exerts no inhibitory effect on IEC-6 cell proliferation at dosages ranging from 1 to 20 μmol/L (Huang et al., 2022).

Moreover, several studies have indicated that ATs can exert effective therapeutic effects in various fibrotic diseases. Experiments conducted using an *in vitro* unilateral ureteral obstruction (UUO) murine model have shown that AT-Ⅰ inhibits myofibroblast phenotype and fibrotic development in the murine kidney by targeting fibroblast-to-myofibroblast differentiation (FMD) as well as EMT (Guo et al., 2021). Research on a bleomycin-induced pulmonary fibrosis model in Sprague-Dawley rats has demonstrated that AT-III mitigates oxidative stress and lung fibrotic damage in the rat pulmonary fibrosis model by activating the Nrf2/NQO1/HO-1 pathway (Huai and Ding, 2020). Current experiments suggest that the upregulation of the Nrf2/NQO1/HO-1 pathway can effectively ameliorate UC, rendering it a potential therapeutic strategy for UC treatment (Amirshahrokhi and Imani, 2023; Ekhtiar et al., 2023). Hence, pending additional validation through research, one mechanism by which AT-III exerts its anti-intestinal fibrosis properties may be the stimulation of the Nrf2/NQO1/HO-1 pathway. These findings collectively indicate that AT-I and AT-III hold promise as potential drugs for the future treatment of UC associated intestinal fibrosis.

### 4.7 Anti-thrombus

In the colonic tissues of both UC patients and experimental UC animal models, platelet activation has been observed, leading to increased thrombus formation by binding to the vascular endothelium. Extraintestinal thrombosis is more common in the DSS-induced UC animal, which also exhibits an increase in serum coagulation markers. Thrombus formation results in ischemic inflammation in the intestinal microvascular system, further exacerbating tissue damage. Furthermore, research has indicated a local increase in procoagulant and prothrombotic events within the microvascular system of UC intestinal tissues, which is associated with subclinical systemic thrombosis in patients. Thrombus formation is a significant complication in inflammatory bowel disease (IBD) patients, accounting for an estimated 25% of IBD-related mortality causes. The risk of thrombotic events is particularly elevated during disease flares and periods of chronic inflammation. Therefore, thrombus formation plays a crucial role in the progression of UC and underscores the importance of early consideration of anti-thrombotic therapy for UC patients (Nelson et al., 2023).

Research has shown that both AT-II and AT-III exhibit significant anti-thrombotic effects. AT-II at a concentration of 10 μmol/L effectively inhibits *in vitro* aggregation of mouse and human platelets stimulated by collagen. This effect is possibly achieved by suppressing the PI3K-Akt pathway, thereby inhibiting platelet activation (Chen et al., 2016a). Furthermore, thromboxane analog (U46619)-induced human platelet aggregation *in vitro* as well as adenosine triphosphate (ATP) production from platelet-dense granules are markedly inhibited by AT-III. The inhibitory effect is concentration-dependent, with the most pronounced inhibition observed at an AT-III concentration of 5 μmol/L. Further investigations suggest that AT-III may inhibit platelet activation by influencing the MAPK and PI3K-Akt pathways, thus mitigating thrombus formation (Chen et al., 2016b).

AT-II and AT-III also significantly reduce the spreading of human platelets on immobilized fibrinogen, delay clot retraction in plasma, increase the time to initial occlusion in the FeCl_3_-induced carotid artery thrombosis model in mice, and prolong bleeding time. Research has additionally revealed that the inhibitory effects of AT-II and AT-III on platelet activation are akin to the aspirin (Chen et al., 2017). Consequently, AT-II and AT-III may be very promising as therapeutic agents for the management of UC that is complicated with thrombus formation, although more thorough investigation is needed to confirm this. Moreover, AT-II and AT-III’s anti-platelet aggregation activities have significant therapeutic implications for vascular thrombotic disorders and offer a possible path for the creation of new anti-platelet medications.

### 4.8 Anti-angiogenesis

Angiogenesis is a hallmark of chronic inflammatory diseases (Britzen-Laurent et al., 2023). Pathological analysis of human and murine colitis tissues reveals significant alterations in the colonic microvascular system, including vascular dilation, congestion, edema, angiogenesis, microvascular occlusion, and the presence of tortuous vessels of varying diameters (Haep et al., 2015). These abnormal vascular changes precede the development of mucosal ulcers and significantly amplify intestinal inflammatory responses (Britzen-Laurent et al., 2023). In the colitis tissues of both mice and humans, vessels formed under the backdrop of chronic inflammation often exhibit an immature phenotype and are frequently associated with excessive thrombosis or vascular constriction (Langer et al., 2019). Therefore, the pathological generation of blood vessels both enhances inflammation and impairs mucosal healing, making it a critical factor in the chronic progression of UC (Sandor et al., 2006).

The application of anti-angiogenic agents is advantageous in the treatment of inflammatory diseases to some extent (Herrera-Gómez et al., 2022). Studies indicate that most quiescent and moderately active IBD patients exhibit good tolerance to anti-VEGF therapy. *In vivo* and vitro experiments based on inflammation models show that AT-I effectively inhibits angiogenesis in chronic inflammation by reducing the expression of NO, TNF-α, IL-1β, IL-6, VEGF and PIGF (Wang et al., 2009b). Research also suggests that AT-III exerts its anti-angiogenic effects through the direct inhibition of endothelial cells *in vitro* and *vivo*. Thus, inhibition of angiogenesis may be one of the mechanisms by which AT-I and AT-III exert their anti-UC effects. However, further validation is required to elucidate their efficacy and mechanisms.

### 4.9 Colitis-associated colorectal cancer

Colitis-associated colorectal cancer (CAC) is a malignant condition of the colon that arises due to recurrent episodes of chronic intestinal inflammation. It represents one of the most severe complications of UC (Beaugerie and Itzkowitz, 2015). Chronic inflammation is recognized as one of the primary factors contributing to the development of cancer in humans, and long-term, irreversible damage to the gastrointestinal structure and function in UC patients elevates the risk of developing colorectal cancer (Wang S. Q. et al., 2019). Previous research has indicated a positive correlation between the incidence of colorectal cancer in Asian UC patients and the duration of their UC. As the duration of the disease increases, the incidence of colorectal cancer gradually rises. CAC differs from sporadic colorectal cancer in terms of its more severe pathological characteristics, worse prognosis, and a greater number of lesions (Terzić et al., 2010). Consequently, the reduction of UC duration, inhibition of inflammation-driven carcinogenesis, and prevention of CAC development play pivotal roles in the prevention and management of CAC.

Besides, AT-I has a pronounced inhibition of colon tumor formation in the AOM/DSS mouse model. *In vivo* and vitro experiments, AT-I treatment effectively suppresses colon tumor volume growth in AOM/DSS-induced mice, significantly reduces the cell viability of human HCT116 and SW480 cells, and induces apoptosis. The mechanism underlying these effects is associated with AT-I inhibiting the expression of NLRP3, Caspase-1, and ASC, as well as the subsequent release of IL-1β (Qin et al., 2021). Previous research has identified SIRT6 as a target effector of AT-I through molecular docking techniques. Further investigation reveals that AT-I enhances the deacetylase activity of SIRT6 in hepatocytes, promoting PPARα transcription and translation, thereby increasing the expression of its target genes to expedite fatty acid oxidation. Simultaneously, AT-I weakens NF-kB-mediated NLRP3 inflammasome formation, macrophage infiltration, and the expression levels of inflammatory cytokines such as TNF-α, IL-6 and IL-1β, thus suppressing hepatic inflammation and steatosis. Additionally, knocking out SIRT6 genes from mouse livers reduced the inhibitory effect of AT-I on NLRP3 inflammasomes and inflammatory responses induced by high-fat diets (Kong et al., 2022). Inflammasomes are believed to mediate host defense against microbial pathogens while maintaining intestinal homeostasis, excessive activation can lead to inflammatory diseases such as CRC (Beaugerie and Itzkowitz, 2015). Therefore, targeting the SIRT6/PPARα/NF-kB/NLRP3 pathway may be a potential way for AT-I to exert its anti-inflammatory and anti-cancer effects, which requires further validation. Studies also suggest that the inhibitory effect of AT-I on CAC is related to blocking Drp1-mediated mitochondrial fission (Qin et al., 2021). Thus, AT-I could serve as a potential effective anti-cancer drug, playing an important role in the treatment of UC and CAC.

Furthermore, research has reported that AT-Ⅰ exerts its anti-colorectal cancer effects through multiple mechanisms, including the inhibition of tumor cell proliferation, induction of cell death, regulation of cancer stemness, and enhancement of cancer cell immunogenicity (Li et al., 2020; Wang et al., 2020; Xu et al., 2021). It holds promise as a potential drug for combating colorectal cancer. AT-Ⅰ has inhibitory effects on various cancer cell types, and studies indicate that it inhibits tumor cells in breast cancer, lung cancer, ovarian cancer, and other malignancies through multiple pathways (Yao et al., 2019). Preliminary clinical trials using AT-Ⅰ purified extract in gastric cancer patients with cachexia suggest that AT-I contributes to alleviating cachexia symptoms (Liu et al., 2008). However, current clinical evidence is insufficient, and further research at the clinical level is required to confirm AT-I efficacy and safety.

**FIGURE 4:**
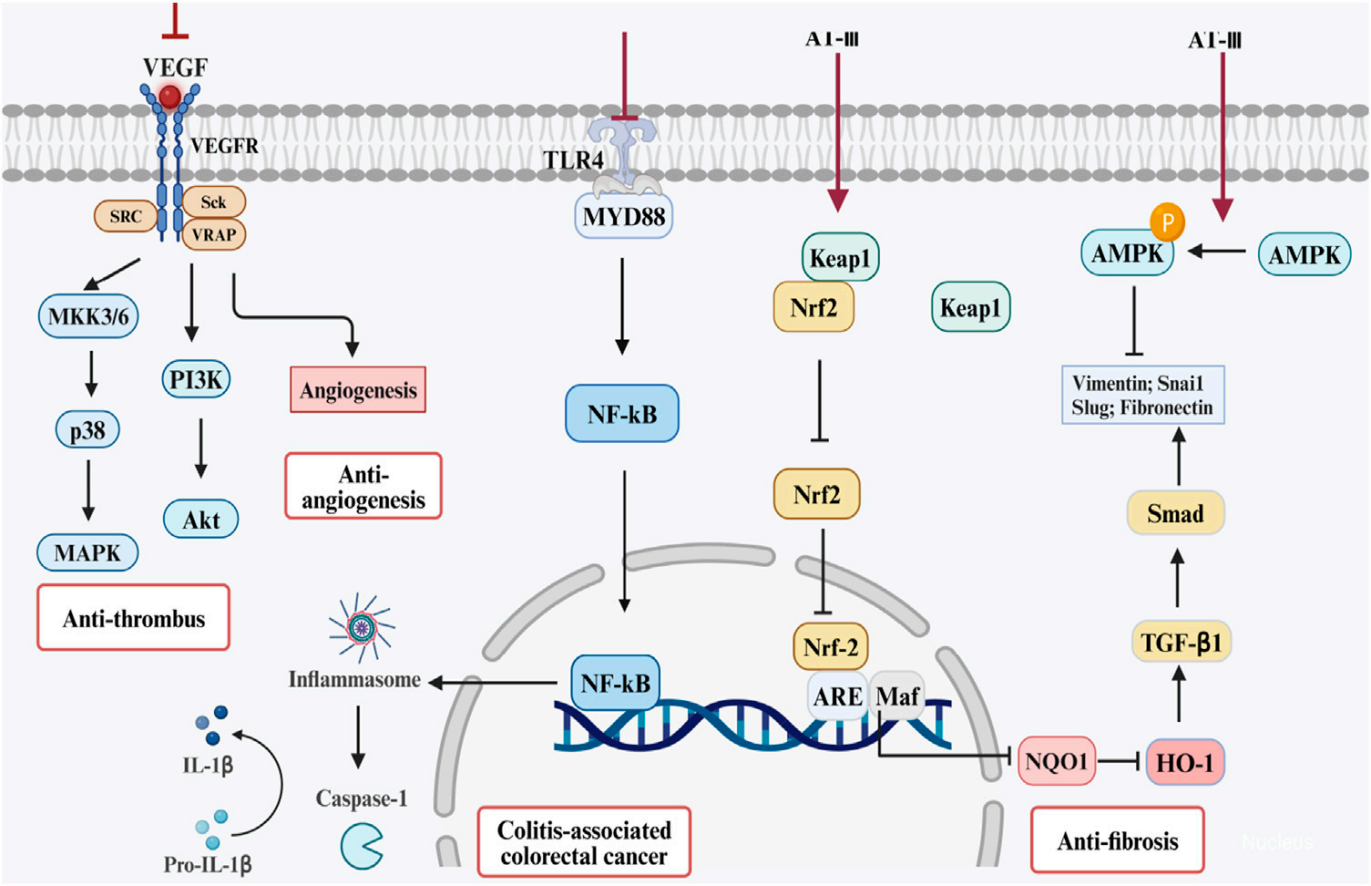
Potential therapeutic mechanisms of ATs for UC and its complications. Note: These effects require validation *in vivo*.

**FIGURE 5:**
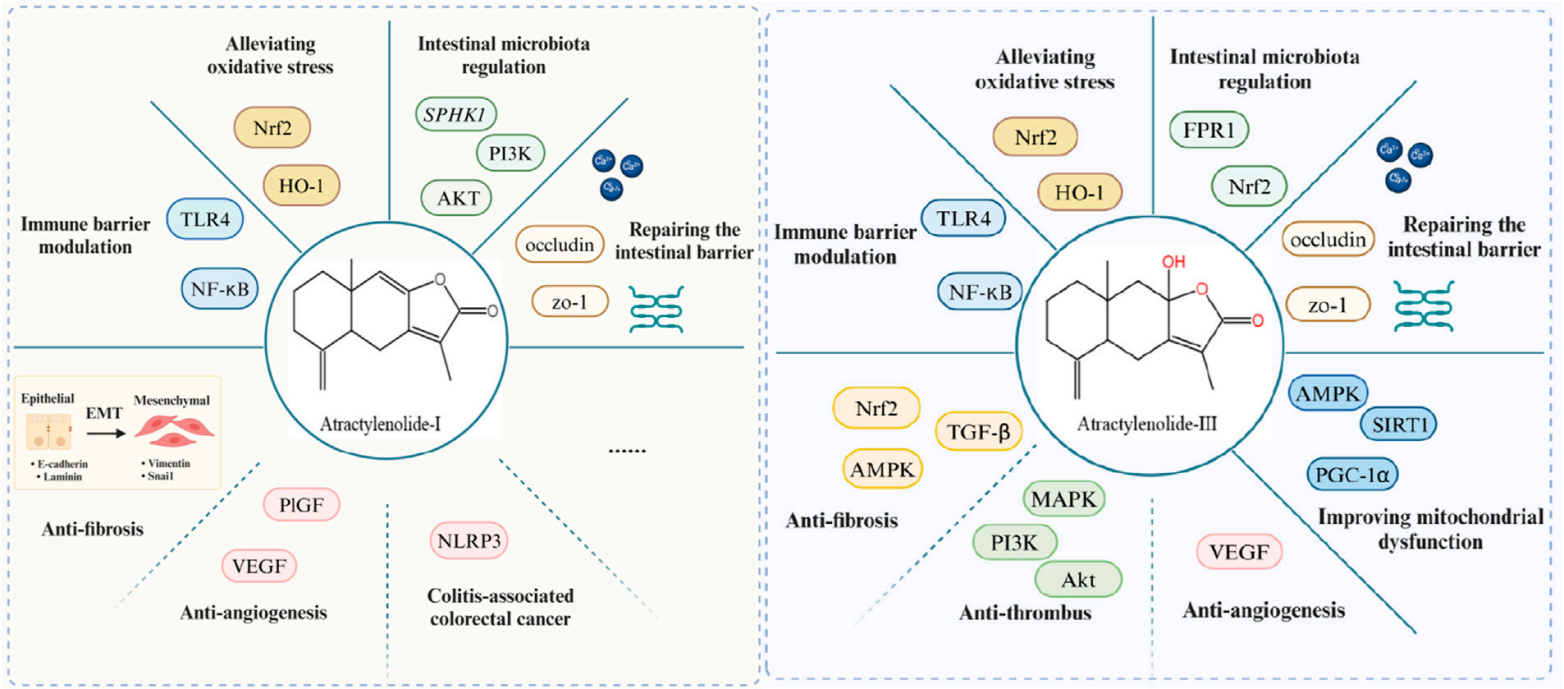
Pharmacologic effects of AT-I and AT-Ⅲ in the treatment of UC and its complications.

AT-II can enhance the sensitivity of cancer cells to drugs. When the concentration of AT-II reaches 150 mg/L, it significantly inhibits the proliferation of colorectal cancer Lovo cells in a dose-dependent manner. In regard to AT-III, studies indicate that AT-III can promote the expression of pro-apoptotic genes such as Bax, caspase-2, and caspase-9 while inhibiting the expression of anti-apoptotic gene Bcl-2. It regulates the Bax/Bcl-2 apoptotic signaling pathway, thus promoting cell apoptosis and significantly inhibiting the growth of HCT-116 tumor xenografts in nude mice (Zhang et al., 2022). Therefore, AT-II and AT-III also hold potential as drugs for combating colorectal cancer, and their role in CAC warrants further research and evidence-based support. (Figures 4, 5).

## 5 Summary and perspectives

The latest findings show that ATs have both similarities and distinctions in their pharmacological effects for treating UC. AT-I and AT-III act as antagonists of the TLR4 receptor on white blood cells, effectively reducing the release of pro-inflammatory factors by blocking the TLR-4/NF-kB/MAPK pathway. This indicates strong anti-inflammatory effects in experiments conducted *in vitro* and *in vivo* (Ji et al., 2014; Ji et al., 2016). Nrf2, a transcription factor, leading to notable antioxidant effects, can be activated by AT-III. Both AT-II and AT-III can trigger the Nrf2/HO-1 pathway to combat oxidative stress and improve colonic inflammation (Ren et al., 2021a). While both AT-I and AT-III can enhance beneficial bacteria abundance and diversity to modulate intestinal microbiota structure, their mechanisms differ slightly (Zhu and Li, 2012; Qu et al., 2022). AT-I, AT-II, and AT-III all promote epithelial cell repair to enhance intestinal barrier function (Song et al., 2017), although any differences in their repair-promoting effects remain unexplored at present. Overall, ATs exhibit promising potential as therapeutic agents for UC by significantly inhibiting its development through various pathways.

Ulcerative colitis (UC) is a chronic, recurrent disease with various complications that increase its clinical severity. Preventing and treating these complications are crucial in managing UC. AT-II and AT-III have significant anti-thrombotic effects (Chen et al., 2016a; Chen et al., 2016b), possibly through inhibiting the activation of the PI3K-Akt pathway. Studies show that patients with IBD have about three times higher risk of venous thromboembolism (VTE) compared to normal individuals, which increases during disease flares. Hospitalized IBD patients without active bleeding during flare-ups are recommended anticoagulant prophylaxis (Nguyen et al., 2014). AT-II and AT-III not only alleviate inflammation in UC but also inhibit platelet activation similarly to aspirin. They hold promise as potential drugs for treating UC and its thrombotic complications. In terms of anti-fibrosis effects, both AT-I and AT-III can inhibit EMT in IEC-6 cells and renal fibrosis through various pathways. Activation of Nrf2 may be one of the targets that AT-III exerts its anti-intestinal fibrosis effects (Huai and Ding, 2020; Guo et al., 2021; Huang et al., 2022). Research on AT-II’s anti-fibrotic properties is lacking currently. Both AT-I and AT-III possess anti-angiogenic effects (Wang et al., 2009b), although their efficacy in treating UC and its complications through this mechanism requires further validation. Notably, studies have shown that ATs inhibit cancer progression by targeting angiogenesis (Yang et al., 2021). Furthermore, CAC is one of the most severe complications in the development of UC, making effective prevention and treatment crucial (Keller et al., 2019). Research shows that post-treatment with AT-I effectively inhibits colon tumor growth in AOM/DSS-induced mice (Beaugerie and Itzkowitz, 2015). Clinical trials using purified AT-I solution suggest it helps alleviate cachexia symptoms in gastric cancer patients (Liu et al., 2008). While the therapeutic effects of AT-II and AT-III on CAC are not yet reported, their significant efficacy against colorectal cancer has been observed *in vitro* and *vivo* experiment (Zhang et al., 2022). Therefore, ATs not only have significant therapeutic effects on UC but also show promising potential in treating its complications, which could reduce the recurrence and improve the cure rate of UC.

Although ATs have advantages in the treatment of UC, their clinical application still has a long way to go. ATs are currently understudied and more studies are still needed to validate the efficacy and mechanisms of ATs in UC and its complications. Additionally, *in vitro* experiments have shown that ATs can be cytotoxic at certain doses (Ji et al., 2014; Huang et al., 2022). Cytotoxicity does not necessarily indicate adverse clinical reactions to the botanical drugs. However, experimental dosages of ATs (ranging from 5 to 50 mg/kg) vary among studies, making it difficult to determine the appropriate dosage range for treating UC. Therefore, further investigation on dosages and duration through controlled variables is necessary to provide guidance for safe clinical application. Lastly, current animal experiment results may not fully represent clinical effectiveness, and high-quality clinical trials are needed in the future to validate the safety and efficacy of ATs.

Furthermore, based on the current research findings of ATs, potential future research directions could be explored. Firstly, there are variations in the therapeutic effects among different ATs, thus, further validation is needed to determine if combining ATs could enhance treatment effects. Secondly, studies have shown that using ATs alone or in combination with cytotoxic drugs can help treat cancer or reduce side effects of radiotherapy and chemotherapy (Bailly, 2021). As the therapeutic advantages of ATs in UC and its complications, they may potentially serve as ideal adjunct medications for other UC treatments in the future, synergistically enhancing efficacy or reducing toxicity. In conclusion, ATs deserve further research in the future.

## Figures and Tables

**Table:** 

**UC**	Ulcerative colitis
**ATs**	Atractylenolides
**CYP450**	cytochrome P450
**Pgp**	P-glycoprotein
**T**_**max**_	peak time
**C**_**max**_	maximum concentration
**AUC**	area under the curve
**Ka**	absorption rate
**ROS**	reactive oxygen species
**T1/2**	half-life
**CLz/F**	clearance rate
**COX-2**	cyclooxygenase-2
**iNOS**	induced NO synthase
**LPS**	Lipopolysaccharide
**NO**	Nitric oxide
**PGE2**	Prostaglandin E2
**TNF-α**	Tumor necrosis factor alpha
**IL-6**	Interleukin-6
**NF-kB**	Nuclear transcription factor-kappa B
**ERK1/2**	extracellular regulated protein kinases1/2
**p38**	p38 mitogen-activated protein kinase
**TLR4**	Toll-like receptor 4
**MyD88**	myeloiddifferentiationfactor88
**MAPK**	mitogen-activated protein kinase
**JNK**	c-Jun N-terminal kinase
**IL-1β**	Interleukin-1β
**INF-γ**	Interferon-gamma
**H4R**	H4 receptor
**TSLP**	thymic stromal lymphopoietin
**IL-8**	Interleukin-8
**TNBS**	Trinitro-benzene-sulfonic acid
**MDA**	malondialdehyde
**CAT**	catalase
**SOD**	superoxide dismutase
**GPx**	glutathione peroxidase
**FPR1**	formy1 peptide receptor 1
**Nrf2**	nuclear factor erythroid 2-related factor 2
**Keap1**	Kelch-like ECH-associated protein 1
**ARE**	anti-oxidant response elements
**HO-1**	heme oxygenase-1
**IEC**	intestinal epithelial cells
**MUC2**	Mucin 2
**zo-1**	zonula occludens-1
**MMP-2**	Matrix metalloproteinases-2
**MMP-9**	Matrix metalloproteinases-9
**PGC-1α**	Receptor-γ coactivator 1-α
**DSS**	Dextran Sulfate Sodium Salt
**SIRT1**	sirtuin 1
**AMPK**	AMP-activated protein kinase
**TGF-β1**	transforming growth factor-β1
**EMT**	epithelial mesenchymal transition
**UUO**	unilateral ureteral obstruction
**FMD**	fibroblast-to-myofibroblast differentiation
**NQO1**	NAD(P)H quinone oxidoreductase-1
**IBD**	Inflammatory Bowel Diseases
**PI3K/Akt**	Phosphatidylinositol 3-kinase/protein kinase B
**U46619**	thromboxane analog
**ATP**	adenosine triphosphate
**VEGF**	Vascular endothelial growth factor
**PIGF**	Placental growth factor
**CAC**	Colitis-associated colorectal cancer
**SIRT6**	sirtuin 6
**AOM**	Azoxymethane
**HCT116**	Human colorectal carcinoma
**SW480**	Human colon adenocarcinoma cells
**NLRP3**	NOD-like receptor thermal protein domain associated protein 3
**ASC**	Apoptosis-associated speck-like protein
**PPARα**	Peroxisome proliferator-activated receptor alpha
**Drp1**	dynamin-relatedprotein 1
**Bcl-2**	B-cell lymphoma-2
**Bax**	BcL2-associated X protein
**VTE**	venous thromboembolism
**SASP**	Sulfasalazine
**PMACI**	phorbol 12-myristate 13 acetate (PMA) and calcium ionophore A23187
